# Timing and Indications for Liver Transplantation for Children with Chronic Liver Disease

**DOI:** 10.3390/children12040449

**Published:** 2025-03-31

**Authors:** Risheka Lakshmi Suthantirakumar, Girish L. Gupte

**Affiliations:** 1 Specialised Foundation Programme in Paediatrics, Imperial College London NHS Hospital Trust, London W2 1NY, UK; 2 Liver Unit (Including Small Bowel Transplantation), Birmingham Children’s Hospital, Birmingham B4 6NH, UK; girishgupte@nhs.net

**Keywords:** paediatric, liver transplantation, biliary atresia, cirrhosis, chronic liver disease, inborn errors of metabolism, cholestasis, pulmonary hypertension, PELD and hepatocellular carcinoma

## Abstract

Chronic liver disease (CLD) in children poses significant challenges, necessitating timely management to mitigate morbidity and mortality. Liver transplantation (LT) has emerged as a transformative intervention, offering improved long-term survival for paediatric patients with CLD. This review explores the evolving landscape of liver transplantation, focusing on indications and timing considerations. The aetiology of CLD is diverse, encompassing intrahepatic, extrahepatic cholestatic conditions, metabolic diseases, malignancy, and drug-induced liver injury. LT is indicated when children exhibit signs of hepatic decompensation, necessitating a comprehensive evaluation to assess transplant suitability. Indications for LT include biliary atresia, inborn errors of metabolism, hepatocellular carcinoma, and emerging indications such as mitochondrial hepatopathies and acute on chronic liver failure. The timing of transplantation is critical, emphasizing the need for early recognition of decompensation signs to optimise outcomes. Advancements in LT techniques and immunosuppressive therapies have enhanced patient and graft survival rates. Various transplant modalities, including reduced-size LT and living-related LT, offer tailored solutions to address the unique needs of paediatric patients. While LT represents a cornerstone in the management of paediatric CLD, careful patient selection, multidisciplinary collaboration, and ongoing refinements in transplant protocols are imperative for optimizing outcomes and addressing the evolving landscape of paediatric liver disease management.

## 1. Introduction

Chronic liver disease (CLD) is defined as the continuing impairment of liver parenchyma over more than six months, leading to fibrosis and eventually cirrhosis of the liver [1,2].

**Figure 1:**
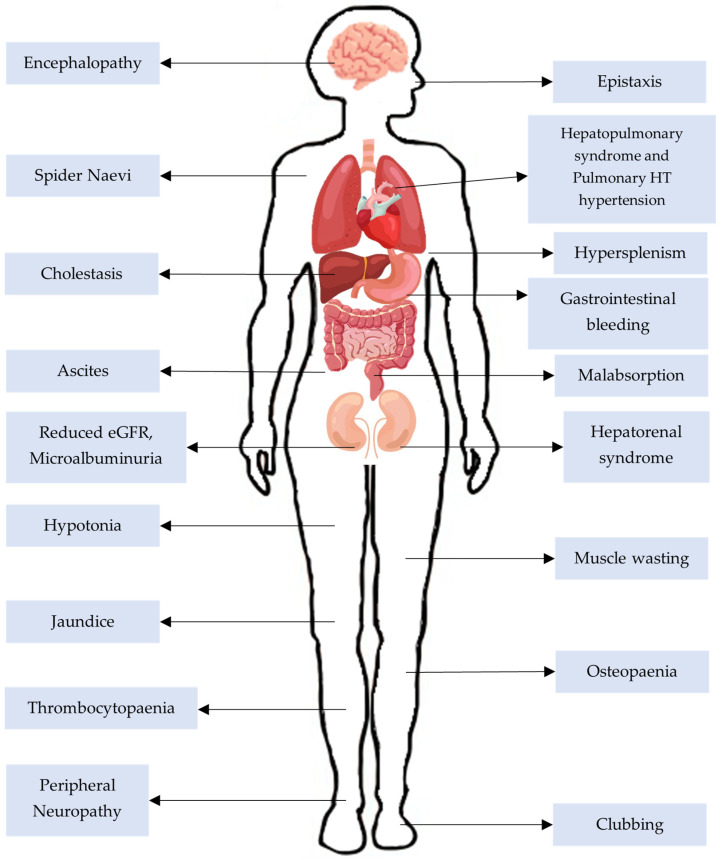
Diagram illustrating the clinical features presented in children with chronic liver disease.

CLD is one of the leading causes of morbidity and mortality in children. In comparison to adults, the clinical features of CLD can develop more rapidly and result in devastating complications (Figure 1) associated with long-term morbidity and mortality [3]. Therefore, appropriate management of this condition is essential to improve long-term survival in children with CLD.

In recent decades, liver transplant has become internationally recognised as providing an opportunity for treating children with chronic liver disease, thus resulting in improved long-term survival [4]. Through constant enhancements of surgical practices, new immunosuppressive medications, and expert clinical management, liver transplantation has transformed the survival rate of paediatric patients with chronic liver disease [5]. The outcomes of liver transplants have advanced exponentially over the last 60 years since the first paediatric liver transplant was conducted by Thomas Starzi at the University of Colorado in March 1963 on a 3-year-old boy with biliary atresia [6]. Although there have been remarkable advances in liver transplantation techniques and long-term management, it can be associated with significant morbidity and mortality risks especially if carried out in the pre-terminal phase. Hence, the timing of transplantation in chronic liver disease is of vital importance [7].

In paediatric patients, signs of hepatic decompensation can be very subtle, such as exhaustion, failure to thrive, and reduced activity level. As children begin to exhibit these signs and symptoms, it is usually the most ideal period for them to be considered for transplantation. It is essential to initiate appropriate treatment before signs of end-stage liver disease begin advancing, which include variceal bleeding and encephalopathy [8].

This article describes the common indications for liver transplantation in chronic liver disease and details the issues surrounding appropriate timing for transplantation.

## 2. Chronic Liver Disease

The main causes of chronic liver disease in younger and older children are presented in Table 1 and Table 2 [4].

## 3. Indications of Liver Transplantation for Children with CLD

In the initial stages of the establishment of liver transplantation that were performed in the 1960s, the most common indication for hepatic replacement was malignancy. However, since the 1980s, with the improvement of immunosuppression agents for these children, liver transplantation has become a more conventional treatment for children with progressive hepatic disease [9,10].

### 3.1. Disease-Specific Indications

#### 3.1.1. Biliary Atresia

The most common type of CLD indicated for liver transplants is biliary atresia. It is an obstructive cholangiopathy of unknown aetiology involving both the intrahepatic and extrahepatic bile ducts, which if left untreated can lead to end stage liver disease This condition presents in neonates several weeks after birth and is clinically seen with persistent jaundice, hepatomegaly, and clay-coloured stools [11]. Although the Kasai procedure, a surgical attempt to restore bile flow, can offer provisional respite, it often fails to prevent long-term liver deterioration in most cases [12]. Approximately 20–30% of paediatric patients with biliary atresia undergo hepatic transplants between the ages of 2 and 18 years after the Kasai procedure [13]. In general, primary hepatic transplantation prior to a Kasai procedure is not indicated in biliary atresia unless they show signs of severe hepatic parenchymal impairment, which can include ascites, coagulopathy, or hypoalbuminaemia [5]. Without timely management, biliary atresia can lead to severe complications such as malnutrition, recurrent cholangitis, and end-stage liver disease, making liver transplantation the definitive treatment to restore normal liver function and improve survival outcomes [14].

#### 3.1.2. Inborn Errors of Metabolism

The second most common indication for liver transplants were inborn errors of metabolism. These include inborn errors of amino acids, carbohydrates, metal, lipid metabolism, and mitochondrial diseases [15].

Metabolic liver diseases can be divided into conditions that cause structural liver impairment such as alpha-1-antitrypsin (AAT) deficiency, Wilson’s disease, and disorders that manifest extrahepatically such as urea cycle disorders, hereditary tyrosinaemia type 1, and glycogen storage diseases.

AAT deficiency is one of the most common indications for liver transplantation amongst the metabolic liver disease to treat the condition. AAT deficiency is a genetic disorder that affects both the lungs and liver due to the lack of AAT, which is a protease inhibitor that prevents destruction of tissue from the neutrophil elastase enzyme [16]. The condition is inherited via an autosomal co-dominant transmission resulting in a mutation of the *SERPINA1* gene. This gene is found in the long arm of chromosome 14 and can lead to abnormal levels of AAT being produced in the liver [17].

Hereditary tyrosinaemia type 1 is an autosomal recessive inborn error of metabolism that affects a child’s liver, kidneys, and peripheral nerves. It manifests due to a deficiency in the fumaryl acetoacetate hydrolase (FAH) enzyme, which is involved in the metabolism of tyrosine [18]. The clinical manifestations of this condition can appear with serious complications including cirrhosis, hepato- and splenomegaly, abnormal blood coagulation, and hypoglycaemia, eventually leading to death in the early months of life [19].

Examples of urea cycle disorders are ornithine transcarbamylase deficiency, carbamylphosphate synthase deficiency, and citrullinemia type I. In severe forms of urea cycle disorders in neonates, liver transplantation should be considered promptly before the development of serious neurological consequences, since these neonates can present with hyperammonaemic coma, often necessitating haemodialysis [20].

Commonly, these conditions can be treated with rigorous dietary constraints and medical supplementation to remove toxic metabolites, to prevent complications [21]. However, it can prove difficult for both patients and their families to adhere strictly to these diets and medical management, and therefore, liver transplantation proves an alternative option to prevent the life-threatening risk of metabolic decompensation and improve quality of life.

### 3.2. Malignancy

Hepatocellular carcinoma (HCC), although rare in children, is the second most common malignant liver tumour. There are a variety of conditions that predispose children to developing cancer; these include perinatally acquired hepatitis B virus, Alagille’s syndrome, hepatorenal tyrosinemia, and various other conditions [22]. Currently, there is no clear evidence suggesting the benefit in survival rate for children with HCC from either neoadjuvant or adjuvant chemotherapy [23]. The foremost curative treatment for HCC is liver transplantation, as it provides the best option for both the widest possible resection of the margins and removing the risk of developing HCC in the diseased sections of the liver [24]. The SIOPEL 1, 2, and 3 studies have also indicated that margin-negative resection is one of the most important factors for enhanced survival rates [16].

### 3.3. Genetic Conditions

There are numerous genetic conditions that present as intrahepatic cholestasis secondary to the loss of an important function in organelles or the small apical domain of hepatocytes [25]. There are several forms with fluctuating clinical features and with a great degree of variability in presentation and prognosis [26]. These comprise Alagille syndrome, progressive familial intrahepatic cholestasis (PFIC), etc.

PFIC is the term used to refer to the collection of rare genetic conditions involving bile acid transporters where a mutation has led to an abnormal level of bile secretion and/or production [27]. In recent decades, there has been an expansion in recognition of many more PFIC subtypes, but the main types include FIC 1 (ATP8B1) deficiency, BSEP (ABCB11) deficiency, and MDR3 (ABCB4) deficiency [28]. Although it is a rare disorder, it can lead to substantial morbidity and mortality. Subsequently, many patients’ symptoms tend to re-emerge or are unmanageable medically or with surgical procedures, such as diverting the bile flow, resulting in many children developing end-stage liver disease. As a result, liver transplant becomes the only option, since it has been found to benefit all three variants of PFIC.

### 3.4. New Indications for Transplantation

Aside from conventional indications such as biliary cirrhosis, acute liver failure, and infectious hepatitis, new indications for transplantations are becoming more prevalent due to more sophisticated peri- and postoperative care [29]. New indications for transplant include mitochondrial hepatopathologies, which were previously believed to be non-transplantable.

### 3.5. Mitochondrial Diseases

Mitochondrial hepatopathies are a group of conditions which can also be due to mtDNA depletion, leading to severe hepatopathy [30], which can comprise epileptic encephalopathy and visual impairment, e.g., Alpers–Huttenlocher syndrome caused by autosomal recessive POLG mutations [31]. Moreover, disorders of mtDNA maintenance include defects in genes such as *MPV17*, which is a mitochondrial inner membrane protein that can lead to hepatocerebral mitochondrial disease manifesting with hypoglycaemia, hypotonia, and reduced feeding [32].

However, due to the systemic nature of the diseases, especially neurological manifestations, it has proven challenging for liver transplant to be a mode of treatment [33].

### 3.6. Acute on Chronic Liver Failure (ACLF)

ACLF is defined as an acute precipitating factor affecting the liver, leading to a sudden deterioration in children, with features such as jaundice and coagulopathy complicated by ascites and hepatic encephalopathy within 4 weeks of presentation in patients with either diagnosed or undiagnosed chronic liver disease or cirrhosis [34]. There are multiple different factors that can precipitate ACLF, with the most common being sepsis and gastrointestinal bleeding. The condition is associated with a high rate of mortality, especially in the first 28 days of presentation, and is also related to other complications leading to extrahepatic organ failure, most commonly renal failure. ACLF is predominantly treated with supportive care; however, patients with hepatorenal syndrome will benefit from liver transplantation. However, patients with renal complications, such as acute tubular necrosis and structural kidney injury leading to permanent renal damage, will benefit from kidney transplantation along with liver transplantation [35].

### 3.7. Systemic Complications of CLD

Paediatric patients should be considered for liver transplantation if there are suggestions of liver failure, deterioration of synthetic function of the liver (i.e., falling albumin and worsening prothrombin time), complications of cirrhosis, or systemic complications of chronic liver disease. Complications of cirrhosis include intractable ascites, encephalopathy, variceal haemorrhage, severe intractable pruritis, recurrent cholangitis, and uncorrected coagulopathy. Systemic complications of CLD include hepatopulmonary syndrome (HPS), portopulmonary hypertension (POPH), and hepatorenal syndrome (HRS) [36].

HPS is defined as a reduction in arterial oxygen due to dilatation of the pulmonary vasculature in the presence of chronic liver disease, congenital portosystemic shunts, or portal hypertension [37]. The hypoxaemia related to HPS is secondary ventilation: perfusion mismatch which is principally due to a diffusion defection in the pulmonary bed, leading to an imbalance of the vasodilators and vasoconstrictors [38]. This condition is associated with a high level of morbidity and mortality due to its implications on various organs such as the lungs and should therefore be diagnosed promptly with appropriate treatment following this [39]. Moreover, medical management for HPS has been found to be largely ineffective, making liver transplantation the definite form of management.

POPH, another serious pulmonary vascular disease [40], is defined as pulmonary arterial hypertension, which is complicated by portal hypertension, with or without chronic liver disease. Portal hypertension is characterised by a significant pressure gradient greater than 5mmHg between the hepatic veins and portal venous system [41]. POPH causes an elevation in shear pressure on the pulmonary circulation due to an increase in cardiac output. At present, the haemodynamic measures for portopulmonary hypertension include the following [42]:

1. Mean pulmonary artery pressure of 20 mmHg or more;
2. Pulmonary artery wedge pressure 15 mmHg or less;
3. Pulmonary vascular resistance 3WU or more.

Patients with POPH commonly present with dyspnoea on exertion, as well as fatigue, peripheral oedema, and ascites [43]. Commonly, portal hypertension will precede by numerous years prior to the development of portopulmonary hypertension [44]. Importantly, children with portal hypertension who are being considered for liver transplant should be screened for portopulmonary hypertension using a transthoracic echocardiography (TOE), especially children with a high index of suspicion [45]. Generally, patients can undergo liver transplant without the necessity for pulmonary hypertension therapies if they have hyperdynamic circulation and volume overload. However, it is a contraindication for children with portopulmonary hypertension to undergo a liver transplant if they have consistently significantly raised pulmonary vascular resistance despite treatment for pulmonary hypertension [46]. It is essential that children with either confirmed portopulmonary hypertension or suspected diagnosis should be referred to a multidisciplinary centre specialising in pulmonary hypertension since the care for these children can be multifaceted.

HRS is a multiorgan disorder that impacts both the kidneys and liver, caused by cirrhosis and portal hypertension that activates the neurohormonal cascade leading to splanchnic and systemic vasodilation from the release of vasodilators [47]. These patients clinically present with oliguria and symptoms of liver failure [48].

### 3.8. Timing and Referral for a Liver Transplantation

The initial step in this extensive process is the evaluation section to recognise the necessity of the transplant by a physician and refer them to a transplant centre based on the child’s signs and symptoms.

#### 3.8.1. The PELD Scoring System

The Paediatric End-Stage Liver Disease (PELD) scoring system is an internationally applied principle which provides a numerical calculation of the risk of mortality to allocate livers for transplantation in paediatric patients [49]. The system is equivalent to the Model for End-Stage Liver Disease (MELD score), another scoring system designed for adults with liver disease. The components of the PELD score include total bilirubin, international normalised ratio for prothrombin time, albumin, age < 1 year, and indication of failure to thrive. The PELD scoring system is calculated using the following formula [50]:

PELD Score = 0.436 (Age (<1 yr.)) − 0.687 × Loge (albumin g/dL) + 0.480 × Loge (total bilirubin mg/dL) + 1.87 × Loge (INR) + 0.667 (Growth failure (<−2 Std. Deviations present))

The PELD scoring system can range from −99 to 99; however, patients generally score between 6 and 40 [51].

However, recently, there have been some issues raised regarding the practice of the PELD scoring system. (i) There has been concern about the potential underestimation of pretransplant mortality risk in paediatric patients, particularly under the age of 2 years or those with metabolic conditions. These patients tend to manifest with no or very low bilirubin or albumin [52]. (ii) Exception requests were commonly utilised, especially in regions where there was an insufficiency of paediatric liver transplant donors. As a result, in 2005, the PELD scoring system was modified to only be used by children under the age of 12 years, and those above this age used the MELD score, since there was rise in competition between adults and children regarding the allocation of a liver transplant [53].

#### 3.8.2. Referral Process

**Figure 2:**
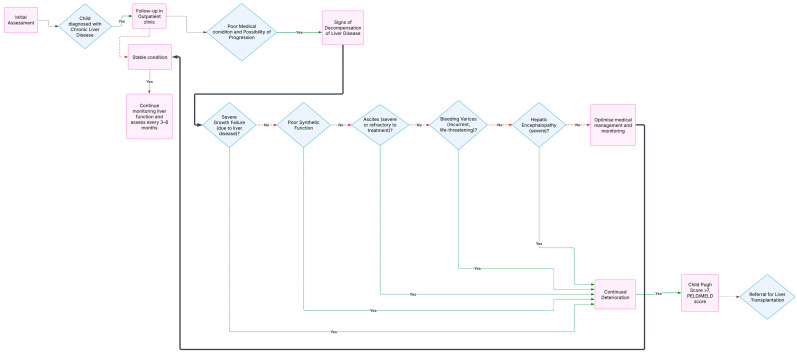
Flowchart illustrating the process for clinical indications and timing for referral of a child with chronic liver disease for management.

Once a child is referred to a transplant centre (Figure 2), there is a rigorous practice which is usually uniform between the centres to assess the suitability of the patient for a transplant and establish a pre and peri-transplantation plan. These can be narrowed to two basic questions. Firstly, are there any alternatives that could be an option for the child? Secondly, does the child have any other co-morbidities that would contraindicate the surgery from taking place or affect the recovery process post-transplantation? A pre-transplantation questionnaire to assess any medical or psychosocial conditions is necessary. Such conditions that may prove a challenge for the surgery include if the child had a severe neurological condition, which prevents them from going under anaesthesia [26].

## 4. Contraindications

Contraindications for liver transplantation can be divided into absolute and relative. Absolute contraindications include extrahepatic malignancy, unrestrained infection external to the hepatobiliary tract, severe cardiopulmonary conditions, or other comorbidities that may affect the survival both during and post-transplantation.

Severe cardiopulmonary disease poses a high risk in patients during liver transplantation, especially a mean pulmonary arterial pressure greater than 50mmHg, since there is a 100% post-procedure mortality rate [54]. Those with a pulmonary arterial pressure of less than 35mmHg are appropriate candidates [27]. Consequently, children with systemic coronary artery, advanced cardiomyopathy, severe valvular heart disease, or ventricular dysfunction are also deemed unfit for a transplantation [54].

Contraindications that may be resolved with time and therefore fall under relative contraindications include poor social support or lack of compliance, especially in older children. In general, LT is not considered an option for paediatric patients if there is a suitable substitute therapy available [28] or if there is damage to other systems other than the liver [55] or if the child has a major systemic infection [56]. Children with impairment of other organs, such as congenital heart disease more profound in patients with biliary atresia, or polycystic kidney disease or congenital hepatic fibrosis, can be a relative contraindication for LT [55]. Therefore, it is essential that thorough cardiac evaluation or of other organ systems are assessed constantly from a very early age to manage these situations [57].

Another relative contraindication is if there is a possibility that the disease will relapse after an LT, such as the risk of a recurring malignancy or infection [58]. Children with primary hepatic malignancy have a relatively large percentage of reappearance of metastatic disease post-LT. Therefore, initial resection and alternative chemotherapy treatments may be a more suitable preliminary preference in comparison to LT.

Nevertheless, due to major enhancement in supportive methods and medical expertise, these relative contraindications have been gradually declining in society. Nonetheless, due to the shortage of donor livers, it is essential that medical professionals appropriately prioritise paediatric patients with utmost need for the liver transplant and who will benefit the greatest from the procedure [59].

## 5. Liver Transplantation

Up until the early 1980s, only whole liver transplants of a donor with a weight as parallel as possible to the recipient were used for paediatric liver transplants [4]. Recent advancements in paediatric liver transplantation have introduced several novel surgical techniques to address the challenges faced in treating children with chronic liver disease. These innovations include split-liver transplantation, which allows a single donor liver to be divided and transplanted into two recipients, thereby increasing the donor pool. Another key technique is reduced-size liver transplantation, where only a portion of a donor’s liver is used, making it possible to transplant livers from larger donors into smaller paediatric patients. Auxiliary liver transplantation, in which a part of the diseased liver is left in place while a healthy graft is transplanted, has been particularly useful in cases where regeneration of the native liver is possible [60]. Moreover, improvements in surgical precision and minimally invasive approaches have reduced complications and improved long-term survival outcomes in paediatric patients. Advanced imaging techniques, such as intraoperative ultrasound, enhance the accuracy of these procedures by providing real-time views of vascular structures. These developments, combined with better postoperative care and improvement in immunosuppression agents, have significantly enhanced survival rates, with one-year survival rates now exceeding 90% for paediatric liver transplants [12,14,61].

## 6. Conclusions

In conclusion, clinicians involved in long-term follow-up of children with chronic liver disease need to evaluate the subtle symptoms and signs that may indicate a decompensated liver function and initiate a prompt referral to liver transplant centre. Liver transplantation is no longer a lifesaving intervention but continues to propose optimism for improved quality of life and long-term survival.

## Figures and Tables

**Table 1:** Causes of chronic liver disease in younger children.

Cirrhosis from CLD	Metabolic-Associated Steatotic Liver Disease
Metabolic Liver Disease	Alpha-1 antitrypsin deficiency
	Cystic fibrosis-associated liver disease
Cholestatic Liver Diseases	Biliary atresia
	Alagille syndrome
	Progressive familial intrahepatic cholestasis

**Table 2:** Causes of chronic liver disease in older children.

Cirrhosis from CLD	Autoimmune Hepatitis
	Primary sclerosing cholangitis
	Budd Chiari syndrome
	Chronic hepatitis B or C viral infection
	Cryptogenic liver disease
Malignant Diseases of the Liver	Hepatocellular carcinoma
	Hepatoblastoma
Metabolic Liver Disease	Wilsons disease
	Hereditary tyrosinemia
	Urea cycle defects
	Glycogen storage disease type IV
	Familial hypercholesterolemia
Cholestatic Liver Diseases	Sclerosing cholangitis
	Idiopathic neonatal hepatitis
	Portal biliopathy with biliary cirrhosis
